# Supplementary material for: Evolution of antibody immunity following Omicron BA.1 breakthrough infection
Source: Nat Commun. 2023 May 12;14:2751. doi: 10.1038/s41467-023-38345-4 (PMC10180619; doi:10.1038/s41467-023-38345-4)
Supplement: Supplementary file 1 — Supplementary Information [file 41467_2023_38345_MOESM1_ESM.docx]

**Supplementary Table 1. Donor Characteristics.**

| **Characteristic** | **Donors (n=6)** |
| --- | --- |
| **Age range** (median) | 19-38 (23) |
| **Sex** |  |
| Female | 5 |
| Male | 1 |

**Supplementary Table 2. Donor vaccination and infection history.**

| **Donor ID** | **IML4042** | **IML4043** | **IML4044** | **IML4045** | **IML4054** | **IML4055** |
| --- | --- | --- | --- | --- | --- | --- |
| Vaccination History | 2x BNT162b2 | 2x BNT162b2 | 2x BNT162b2 | 2x BNT162b2, 1x mRNA-1273 | 3x mRNA-1273 | 3x BNT162b2 |
| Date of 2nd vaccination dose | 22-Jul-21 | 23-May-21 | 10-Feb-21 | 15-May-21 | 5-May-21 | 1-May-21 |
| Date of 3rd dose (if applicable) | - | - | - | 20-Dec-21 | 11-Dec-21 | 9-Dec-21 |
| Date of infection | 4-Jan-22 | 30-Dec-21 | 2-Jan-22 | 6-Jan-22 | 19-Jan-22 | 6-Jan-22 |
| Days between infection and first (T1) sample collection | 21 | 26 | 23 | 19 | 14 | 27 |
| Days between infection and second (T2) sample collection | 170 | 139 | 139 | 168 | 122 | 168 |

**
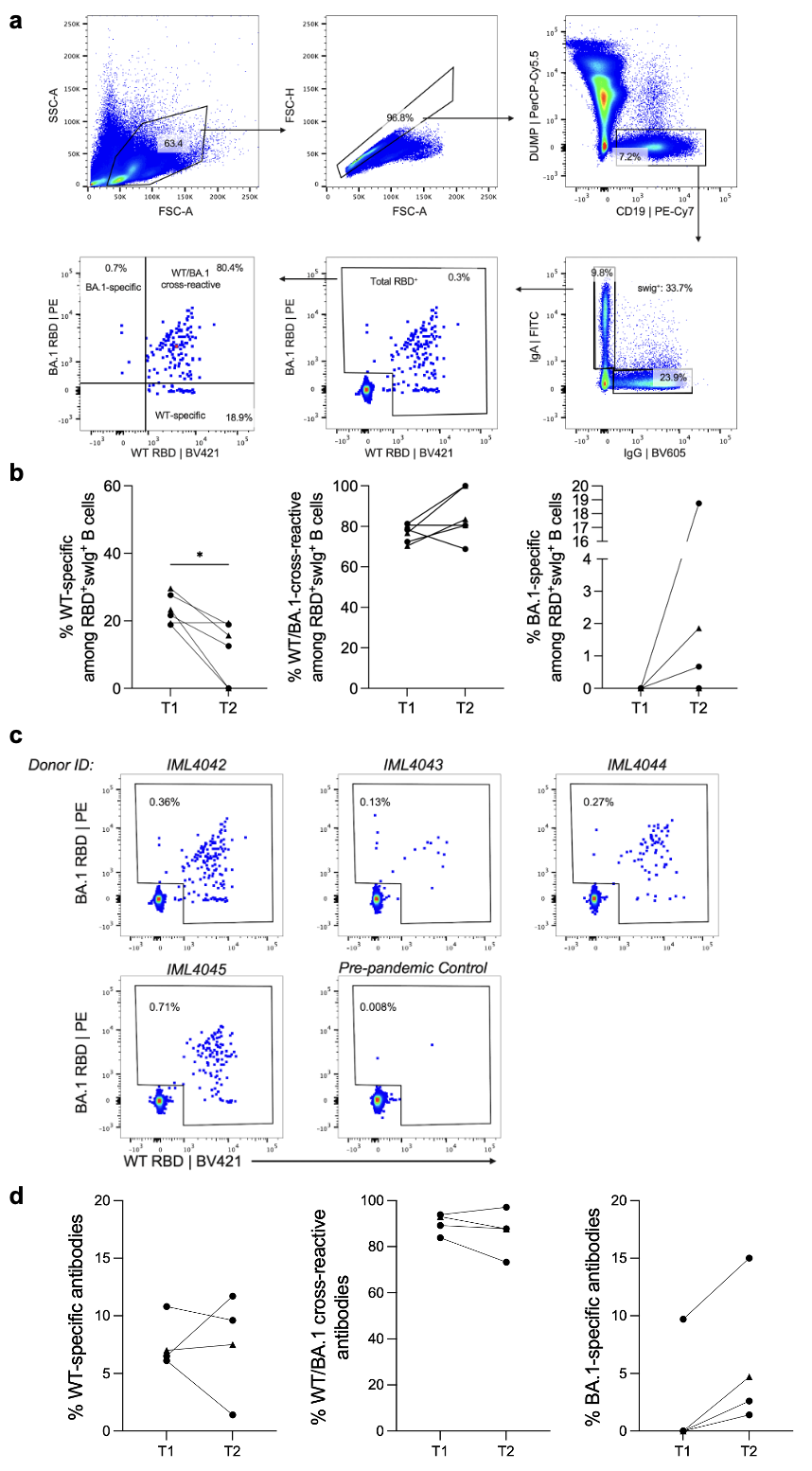
**

**Supplementary Fig. 1. Cross-reactivity of RBD-directed B cells at early and late time points following BA.1 breakthrough infection. a,** Representative FACS gating strategy to determine frequencies of WT and/or BA.1 RBD-reactive B cells among class-switched (IgG^+^ or IgA^+^) B cells. The frequency of events in each gate relative to the parent gate are shown as percentages in each plot. **b,** Proportion of RBD-directed class-switched B cells that are (left) WT-specific (statistical *P* = 0.015), (middle) WT/BA.1 cross-reactive, and (right) BA.1-specific at 1-month (T1) and 5-6-month (T2) time points, as determined by flow cytometry. Donors infected after two-dose mRNA vaccination (n = 4) are shown as circles and those infected after a third mRNA booster dose (n = 3) are shown as triangles. **c,** FACS gates used for single-cell sorting of WT and/or Omicron BA.1 RBD-specific memory B cells in 4 individuals 5-6 months following BA.1 breakthrough infection. Donors IML4042, IML4043, and IML4044 experienced breakthrough infection following two-dose mRNA vaccination, and IML4045 was infected after a third mRNA dose. A healthy pre-pandemic donor sample is shown as a control. **d,** Proportion of BA.1-specific, WT-specific, and WT/BA.1 cross-reactive antibodies isolated from breakthrough infection donors (n = 4) at 1-month^12^ (T1) and 5-6-month (T2) time points, as determined by BLI. Connected data points represent paired samples for each donor. Statistical comparisons were determined by two-sided Mann-Whitney U tests. ***P* < 0.01. FSC-A, forward scatter area; FSC-H, forward scatter height; swIg^+^, class-switched immunoglobulin; SSC-A, side scatter area.


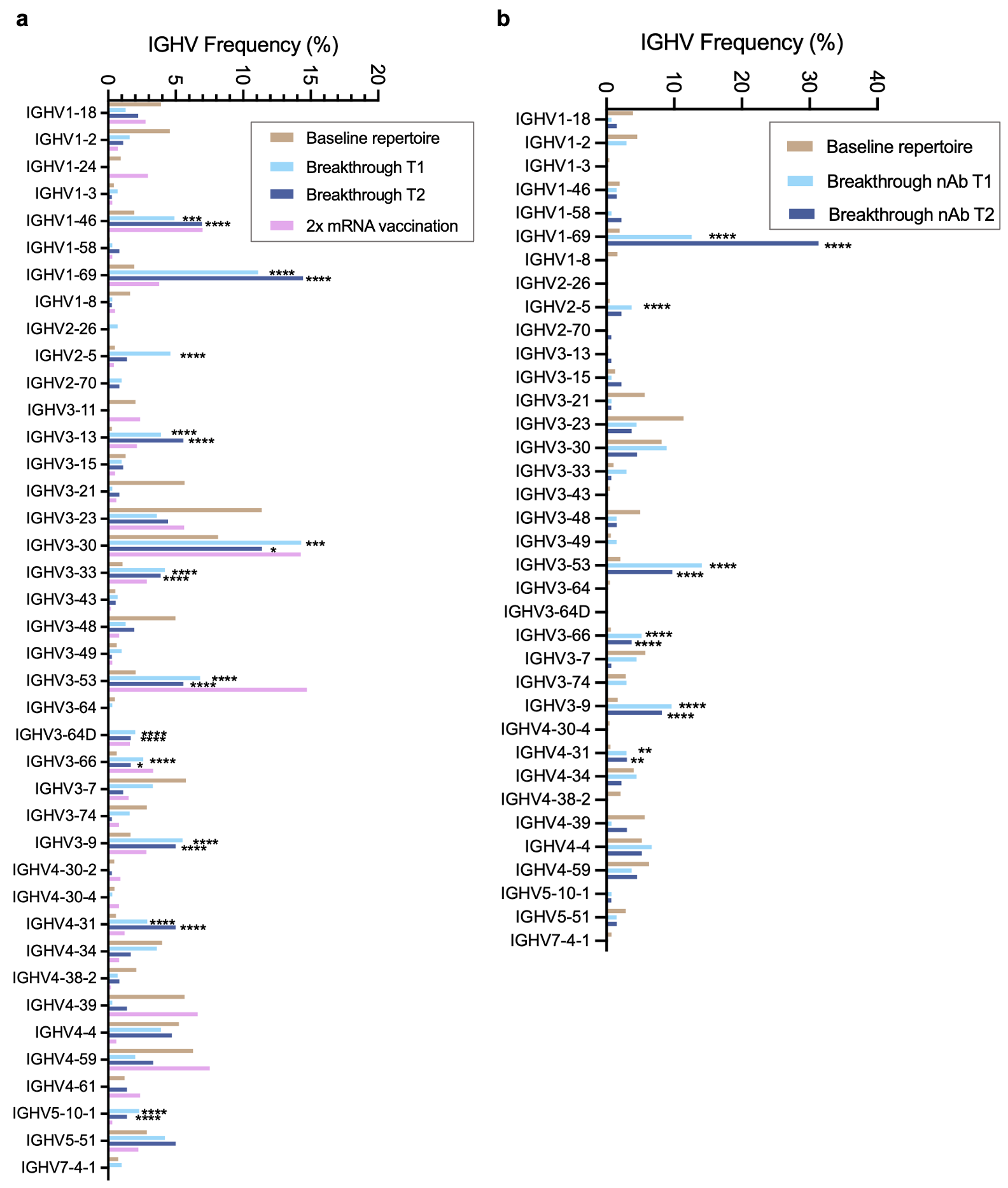


**Supplementary Fig. 2. IGHV germline usage among cross-reactive and cross-neutralizing antibodies.** Human IGHV germline gene usage frequencies among (**a**) WT/BA.1 cross-reactive antibodies and (**b**) D614G/BA.1 cross-neutralizing antibodies isolated 1-month^12^ (T1) and 5-6 month (T2) following breakthrough infection. Germline gene distribution of RBD-directed antibodies derived from two-dose mRNA-vaccinated/uninfected donors were obtained from the CoV-AbDab database ^40^. Human baseline (unselected) repertoire frequencies were included for reference ^39^. Statistical comparisons were made by two-sided Fisher’s exact test compared to the baseline repertoire. IGHV, immunoglobulin heavy variable domain. **P* < 0.05, ***P* < 0.01, ****P* < 0.001, *****P* < 0.0001. Source data and statistical details are provided as a Source Data file.


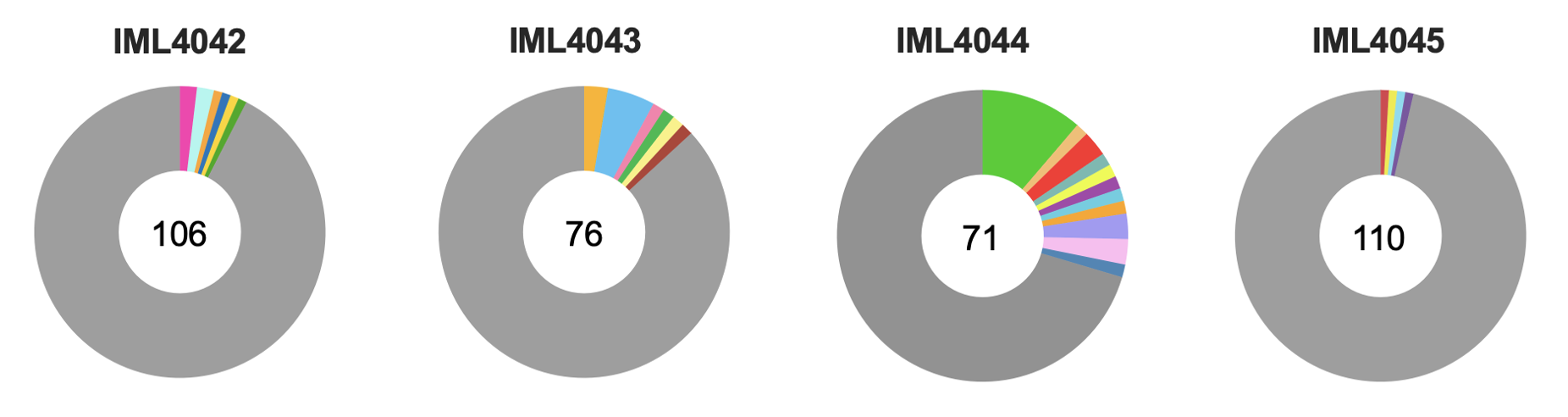


**Supplementary Fig. 3. Persistent clonal lineages observed at early and late time points following BA.1 breakthrough infection.** Proportion of antibodies isolated at the late time point that are clonally related to antibodies isolated at the acute time point. Each colored slice represents a distinct persistent clonal lineage (defined as antibodies with the same heavy and light chain germlines, same CDR3 lengths, and > 80% CDRH3 sequence identity). Antibodies belonging to lineages observed only at the 5-6-month time points are combined into a single gray segment. The total number of antibodies are shown in the center of each pie. Source data are provided as a Source Data file.


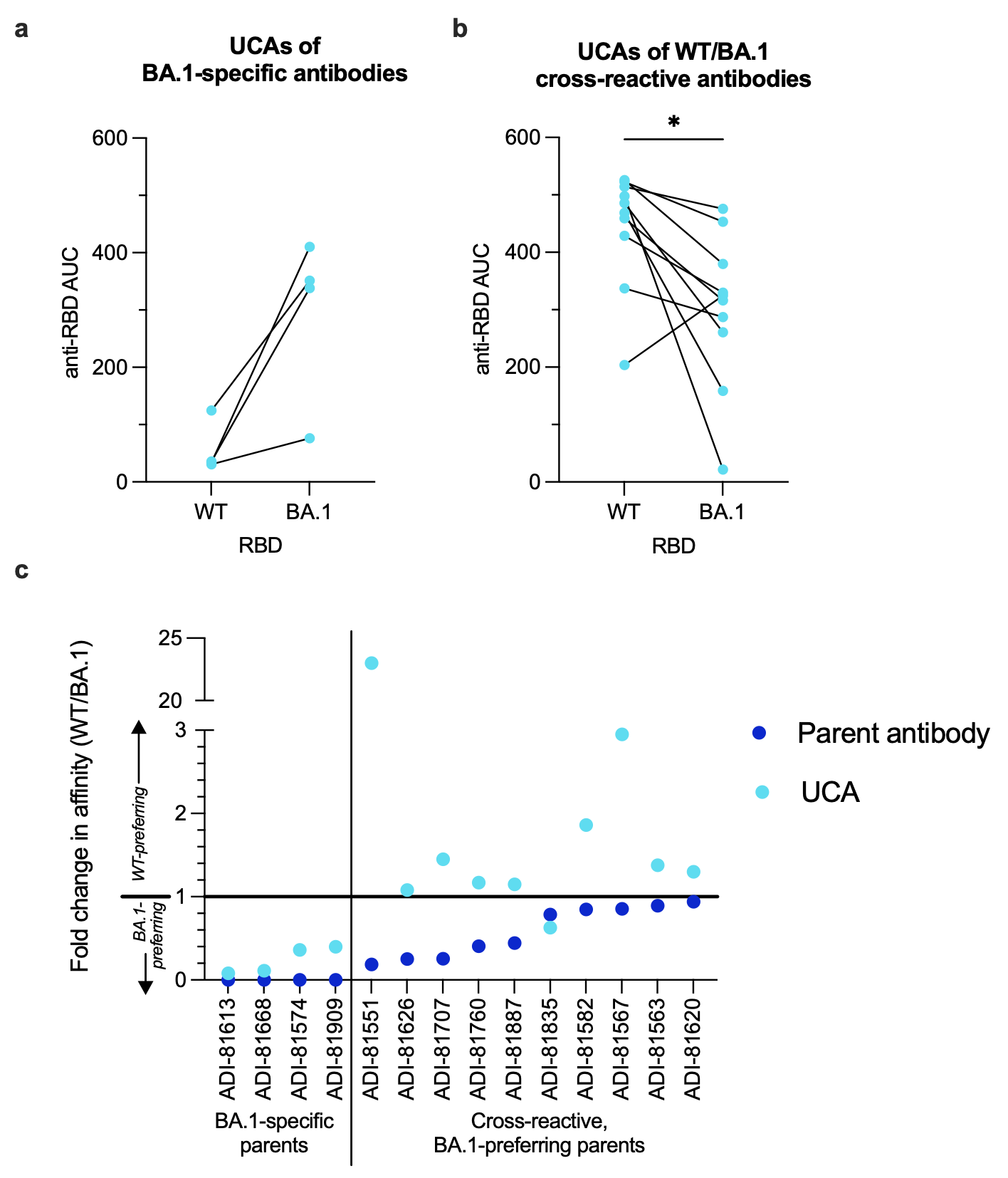


**Supplementary Fig. 4. ELISA binding of unmutated common ancestors of BA.1/WT cross-reactive and BA.1-specific antibodies isolated 5-6 months following BA.1 breakthrough infection. a-b,** Binding of unmutated common ancestor antibodies derived from (**a**) BA.1-specific antibodies (n = 4) and **(b)** WT/BA.1 cross-reactive antibodies (n = 10) that displayed preferential binding to the BA.1 RBD. **c,** Fold change in binding affinity for each parent antibody (dark blue) and UCA (light blue) pair. A fold change greater than one indicates stronger binding to WT relative to BA.1 RBD, and those less than one indicates stronger binding to the BA.1 RBD. Statistical significance was determined by two-sided Wilcoxon matched-pairs signed rank test (*P* = 0.125 and 0.0195 for BA.1-specific and WT/BA.1 cross-reactive antibodies, respectively). AUC, area under the curve. Source data are provided as a Source Data file.

**
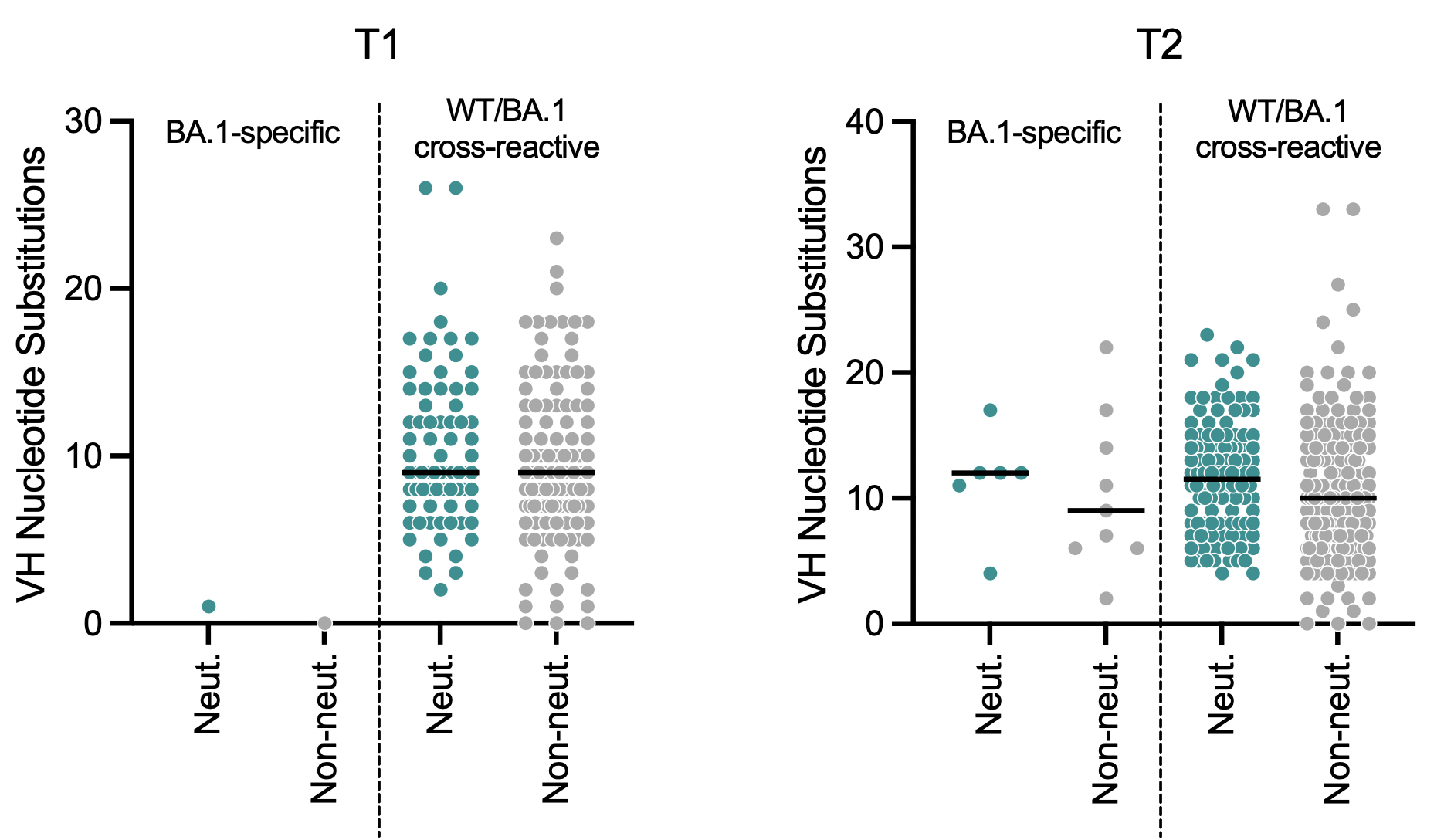
**

**Supplementary Fig. 5. Somatic hypermutation levels of neutralizing and non-neutralizing antibodies isolated following breakthrough infection.** SHM levels, as determined by the number of nucleotide substitutions in the variable heavy (VH) region, at the early^12^ (T1; left) and late (T2; right) time points among neutralizing and non-neutralizing WT/BA.1 cross-reactive (n = 74 neutralizing and 111 non-neutralizing antibodies at T1; n = 118 neutralizing and 167 non-neutralizing antibodies at T2) and BA.1-specific antibodies (n = 1 neutralizing and 1 non-neutralizing antibody at T1; n = 6 neutralizing and 9 non-neutralizing antibodies at T2). Antibodies that display IC_50_s <2 µg/ml are defined as neutralizing. Medians are shown by black bars. Median SHM levels were compared via Kruskal-Wallis analysis and determined to be not significantly different at both time points. Source data are provided as a Source Data file.

**
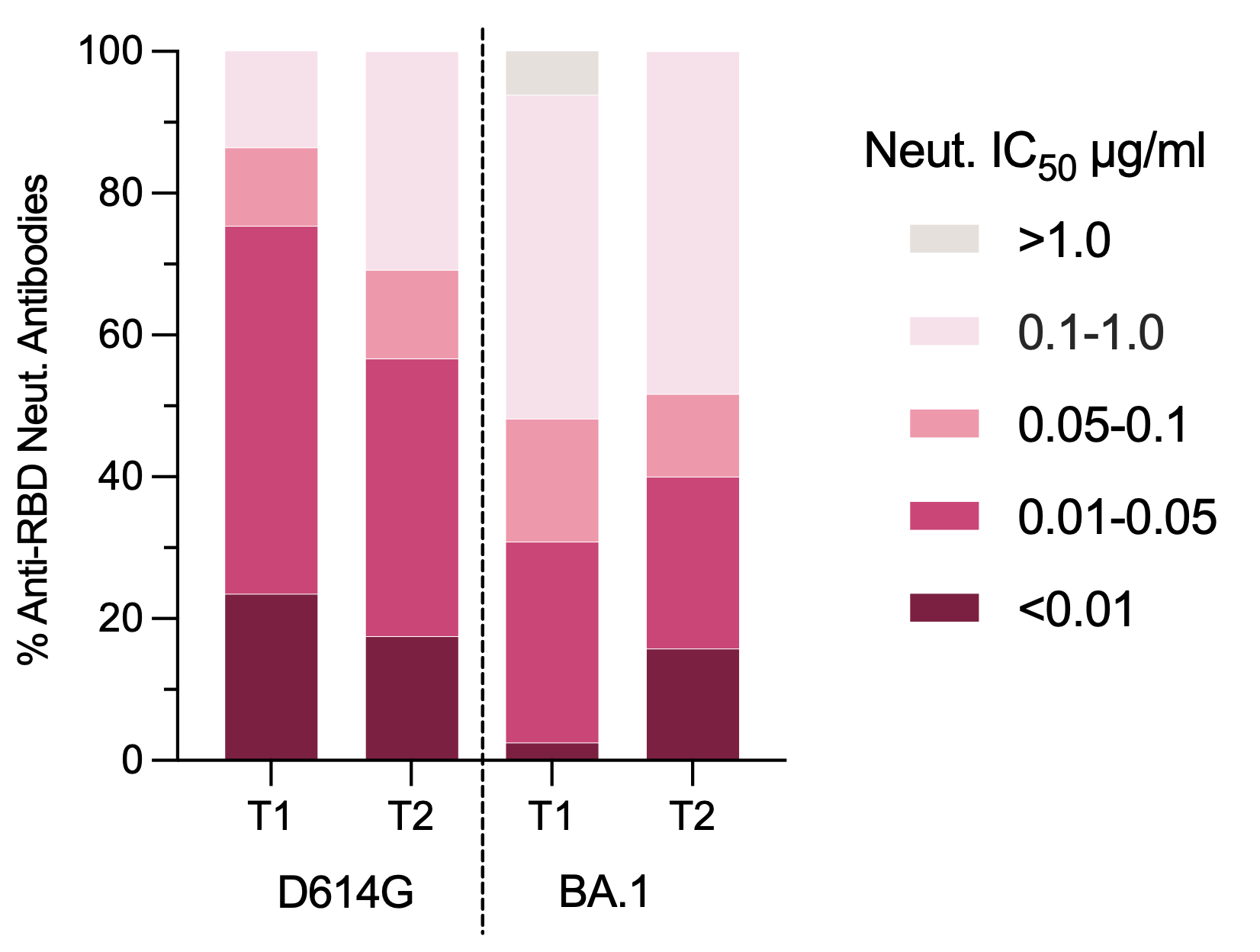
**

**Supplementary Fig. 6. Neutralization potency of D614G/BA.1 cross-neutralizing antibodies isolated at early and late time points following BA.1 breakthrough infection.** Proportion of antibodies isolated at the early^12^ (T1) and late (T2) time points with the indicated neutralization IC_50_s against SARS-CoV-2 D614G and BA.1, as determined by MLV-based pseudovirus neutralization assay. Only cross-neutralizing antibodies, defined as those with IC_50_s <2 µg/ml, are shown. Statistical comparison of BA.1 neutralizing activity by the top ten percentiles of antibodies isolated at early and late time points show significantly more potent neutralization by antibodies identified at the late time point (*P* = 0.0004). Statistical significance was determined by bootstrapping analysis of 10^th^ percentile difference using 5,000 bootstrap iterations, and *P*<0.0001). Source data are provided as a Source Data file.


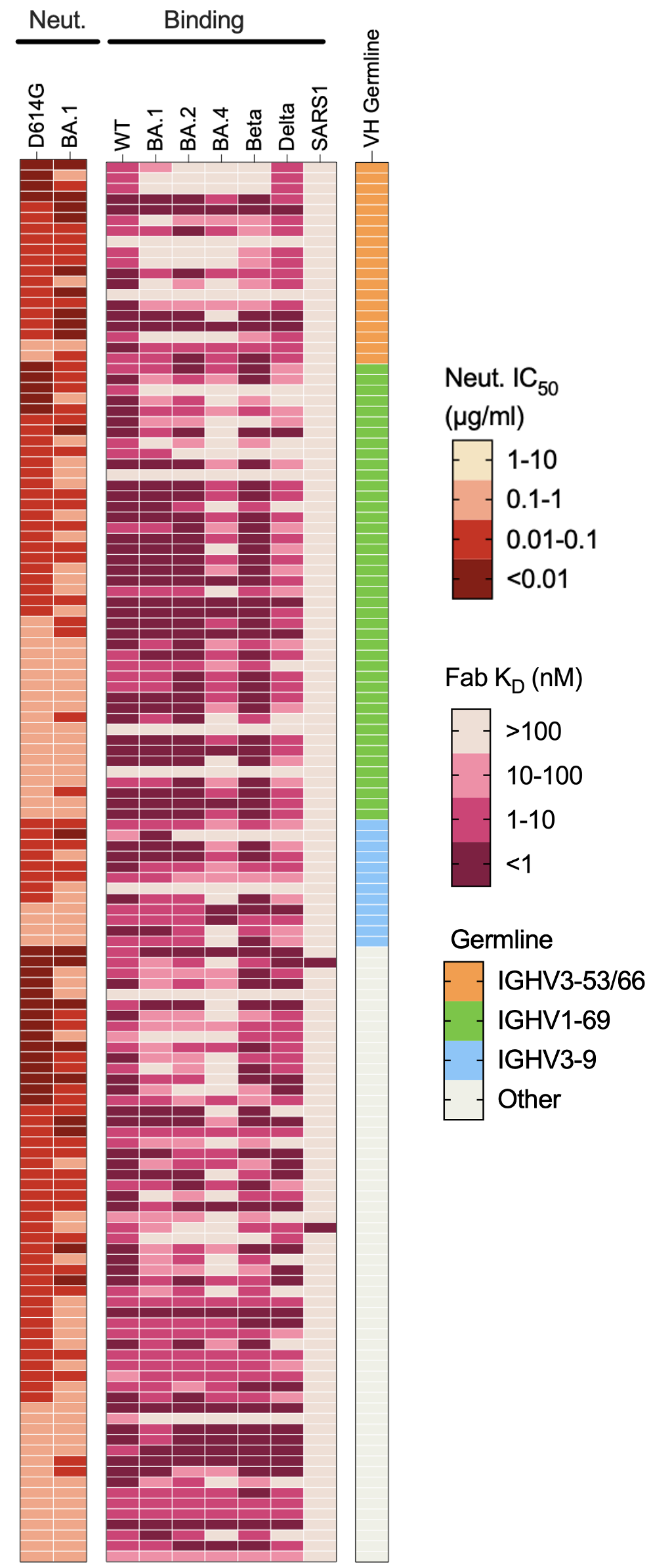


**Supplementary Fig. 7. Binding breadth of D614G/BA.1 cross-neutralizing antibodies**. Heatmap showing neutralization IC_50_s and SARS-CoV-2 variant RBD binding affinities of D614G/BA.1 cross-neutralizing antibodies isolated 5-6 months following BA.1 breakthrough infection**.** Antibodies utilizing convergent germline are indicated in the right-most column. Source data are provided as a Source Data file.


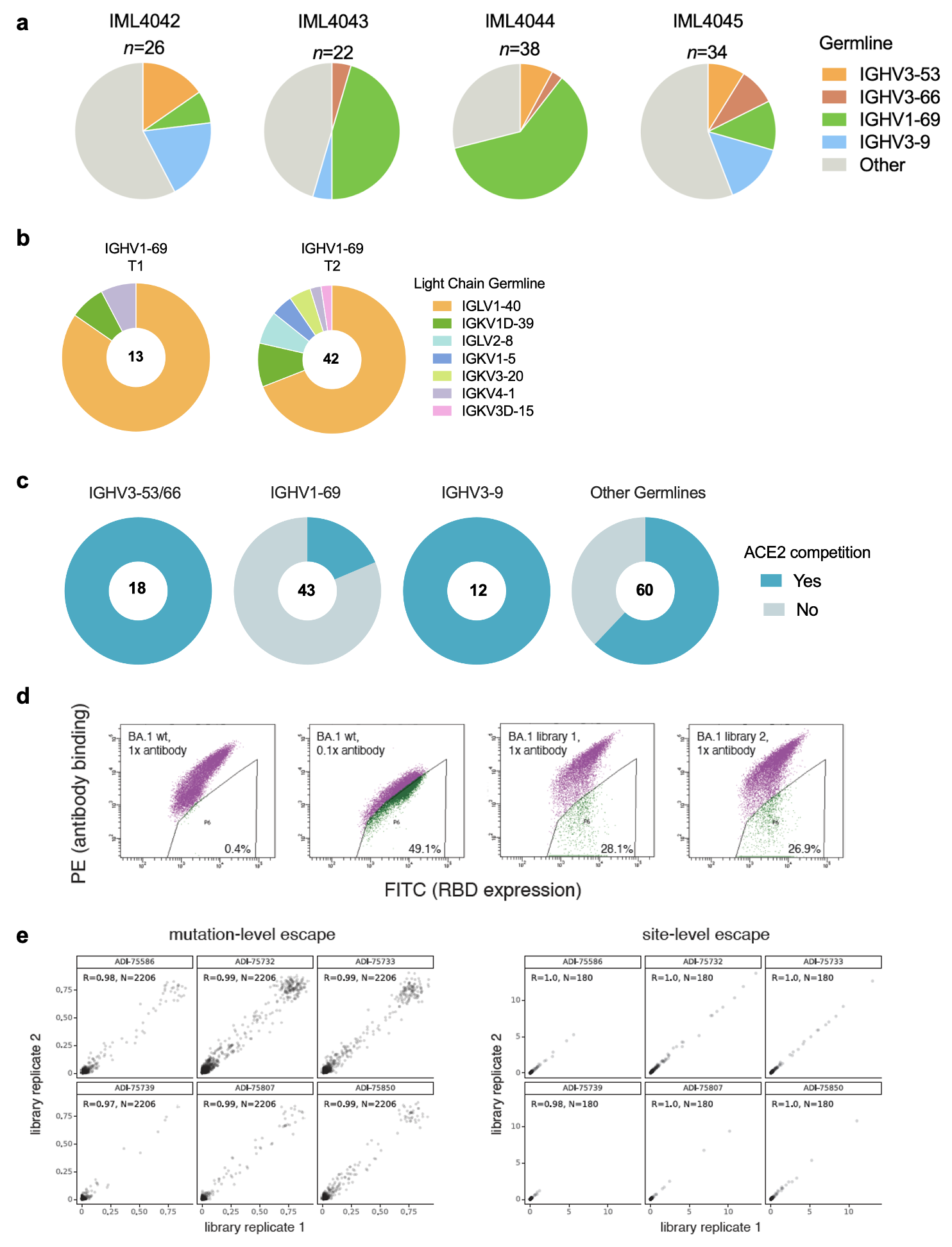


**Supplementary Fig. 8. Sequence and binding features of antibodies utilizing convergent germline genes. a,** Pie charts showing the proportion of cross-neutralizing antibodies isolated from each donor that utilize convergent germline genes. The total number of antibodies isolated from each donor is indicated above each pie chart. **b,** Pie charts showing light chain germline usage among *IGHV1-69* antibodies isolated at 1-month^12^ (T1) and 5-6 month (T2) time points. The number of antibodies analyzed from each time point is indicated in the center of each pie. **c,** Proportion of cross-neutralizing antibodies utilizing the indicated germline genes that compete with the ACE2 receptor for binding, as determined by a BLI competition assay. The number of antibodies analyzed is shown in the center of each pie. **d,** Representative FACS gates used to select antibody-escape mutations in yeast-displayed Omicron BA.1 mutant libraries. Gates were drawn to capture ~50% of wildtype Omicron BA.1-expressing yeast labeled at an antibody concentration 0.1x the selection concentration. From duplicate mutant libraries, yeast cells in the antibody-escape bin were sorted and sequenced. Post-sort mutant frequencies were compared to the pre-sort population to calculate per-mutant “escape fractions”, the fraction of cells expressing a mutation that were found in the antibody-escape sort gate. **e,** Correlation in per-mutation (left) and per-site (right) escape fractions in replicate library selections for each antibody. Source data are provided as a Source Data file.

**References:**

40.   Raybould, M. I. J., Kovaltsuk, A., Marks, C. & Deane, C. M. CoV-AbDab: the coronavirus antibody database. *Bioinformatics* **37**, 734–735 (2021).
